# The Effects of Using Pineapple Stem Starch as an Alternative Starch Source and Ageing Period on Meat Quality, Texture Profile, Ribonucleotide Content, and Fatty Acid Composition of Longissimus Thoracis of Fattening Dairy Steers

**DOI:** 10.3390/foods10102319

**Published:** 2021-09-29

**Authors:** Chanporn Chaosap, Katatikarn Sahatsanon, Ronachai Sitthigripong, Suriya Sawanon, Jutarat Setakul

**Affiliations:** 1 Department of Agricultural Education, Faculty of Industrial Education and Technology, King Mongkut’s Institute of Technology Ladkrabang, Bangkok 10520, Thailand; 2 Department of Animal Technology and Fishery, Faculty of Agricultural Technology, King Mongkut’s Institute of Technology Ladkrabang, Bangkok 10520, Thailand; katatikarnnamwa3@gmail.com (K.S.); ronachai.sit@kmitl.ac.th (R.S.); ksejutar@gmail.com (J.S.); 3 Department of Animal Science, Faculty of Agriculture at Kamphaeng Saen, Kasetsart University, Nakhon Pathom 73140, Thailand; agrsusa@ku.ac.th

**Keywords:** starch source, Holstein steers, meat characteristics, meat flavour, fatty acid profile

## Abstract

The effects of different starch sources (ground corn (CO), ground cassava (CA) and pineapple stem starch (PI)) and ageing period (14 and 21 days) on meat characteristics of Holstein steers were investigated. Starch sources had no effect on meat characteristics, while meat aged for 14 days had less thawing loss than that aged for 21 days. Meat from steers fed PI had higher levels of inosine monophosphate (IMP) than the others (*p* < 0.05). With increasing duration of ageing, the content of IMP and guanosine monophosphate in the meat decreased, while the content of hypoxanthine increased (*p* < 0.05). Meat from steers fed CO had the highest oleic acid but the lowest erucic acid (*p* < 0.05) in contrast to meat from steers fed PI, which had the lowest oleic acid but the highest erucic acid. Steers fed CO appeared to produce healthier meat as this was positively associated with monounsaturated fatty acid content. Meat from steers fed PI had higher levels of IMP, which may be associated with good taste.

## 1. Introduction

Pineapple is one of the most important economic crops in the world, and Thailand is one of the countries with a large pineapple production. In 2020, 1.39 million tons of pineapple fruit were produced, ranking sixth in the world for pineapple production [1]. Pineapples are usually consumed fresh or as pineapple juice, pulp or canned. By-products from pineapple production and canneries can account for 70–75% (*w*/*w*) of the crop, including peel, crown, core, stem and leaf [2]. For economic reasons, the large quantities of by-products from pineapple production should be properly utilized. Otherwise, improper waste management would lead to long-term environmental deterioration, especially through large-scale land clearing, which usually involves biomass burning and soil and water pollution.

In intensive production systems, strategic nutritional supplementation may be undertaken to compensate for deficiencies in the quantity and quality of pasture or forage fed to cattle prior to finishing [3]. High-concentrate commercial cattle fattening systems can provide a sustainable and adequate supply of live cattle to meet domestic consumption requirements with excellent meat quality [3,4,5]. High-concentrate fattening is easy to operate, and the results are predictable [5,6]. The main problem with high-concentrate finishing is the high cost and it is only profitable if the starting value of the animal is low, concentrate costs are low or there is excellent carcass and meat quality [3,5,7]. Male dairy calves that are not desirable for dairy production can therefore be used for beef production, with lower initial cost of animals compared to beef breeds such as Charolais crosses, which are popularly fattened for premium beef in Thailand [8]. However, dairy breeds require 20% more energy and 25% more feed than beef breeds [9]. To achieve cost advantages, agro-industrial feed by-products can be used as alternative feed sources instead of using a main feed source of both roughage and concentrate. Several studies suggest that pineapple by-products such as pineapple cannery by-products [10], pineapple stem [11], pineapple stem by-product silage [12] and ensiled pineapple waste [2] can be used as a promising roughage source to improve rumen function and production performance of animals without compromising production quality and also reduce feed costs. In the past, the pineapple stem was left as waste after replanting before being dried in the sun and burned. Recently, the pineapple stem has been used to extract the enzyme bromelain [13] which can be used as a potential phytomedicinal agent, so the rest of the by-product can be used as a potential starch source for cattle. Pineapple stem starch has similar starch content, but higher amylose content and smaller starch particle size compared to corn and cassava starch: 98, 101 and 100% (*w*/*w*) for starch content; 34.4, 16.2 and 15.3% (*w*/*w*) of whole sample for amylose content; and 9.7, 15.8 and 15.8 µm for starch particle size of pineapple stem, corn and cassava starch, respectively [14]. Khongpradit et al., 2020 [15] found that pineapple stem starch can be a useful starch source with cost advantages for beef cattle to improve growth performance and increase rumen fermentation when formulated as a 40% concentrate compared to other starch sources, ground corn and ground cassava. In addition, Khongpradit et al. (in press) [7] found that the use of pineapple stem starch as a starch source in the concentrate for 206-day fattening dairy steers had a lower feed cost per gain compared to ground cassava and ground corn (2.90, 3.62, and 4.02 USD/kg, respectively).

The most important principles for the palatability of meat are tenderness, juiciness, and flavour, all of which are related to consumer satisfaction. Tenderness was the most important factor influencing beef palatability, but previous research has shown that when tenderness is adequate, flavour is the most important aspect of consumer satisfaction [16]. In addition, the nutritional value of meat, such as fatty acid composition, was an important preference factor for health-conscious customers [17]. Monounsaturated fatty acids (MUFA) are considered better for health, while saturated fatty acids (SFA) can cause health problems [18]. Concentrate-fed beef had more tender meat, higher intramuscular fat with higher MUFA, less connective tissue, and higher palatability scores compared to grass-fed beef [4]. Previous studies have shown that high intramuscular fat can improve flavour, juiciness, and tenderness [4]. Meat flavour is also related to the content of ribonucleotides, such as inosine monophosphate (IMP) and guanosine monophosphate (GMP), as they have an umami flavour [19,20]. However, hypoxanthine, the degradation product of IMP during post-mortem ageing, can cause a bitter taste. Apart from the feeding regime, post-mortem ageing may also serve to improve the tenderness of the meat due to post-mortem proteolysis by specific endogenous enzymes, especially calpains [21], which yield specific protein degradation products such as the 30 kDa degradation product of troponin T [22].

Due to the limited information on the effect of pineapple stem starch in cattle concentrate diet on meat characteristics, the objective of this study was to investigate the effect of pineapple stem starch and also ageing duration on meat quality, texture profile and ribonucleotide content of dairy steers. In addition, the effect of pineapple stem starch on fatty acid composition was investigated.

## 2. Materials and Methods

### 2.1. Animal Ethics

Muscle samples used in this study were from 36 Holstein steers that were part of a study funded by the Agricultural Research and Development Agency of Thailand (ARDA) (project code PRP6205031930), Khongpradit et al., 2020 and Khongpradit et al., (in press). The pineapple stem starch used in this study was obtained from Hong Mao Biochemicals Co, Ltd. in Rayong, Thailand. The Animal Use and Ethics Committee of Kasetsart University, Thailand approved the experimental procedure (ACKU62-AGK-007).

### 2.2. Experimental Cattle and Muscle Collection

Thirty-six Holstein steers with an average weight of 453 ± 35.3 kg and age of 22 months were divided into three treatment groups of 12 steers each and each steer was fed individually. The treatments were three different sources of starch in the concentrate: ground corn (CO), ground cassava (CA), and pineapple stem starch (PI), all at a concentration of 40% (Table S1). From day 1 to day 74, all experimental animals were fed the concentrate ad libitum with Napier grass silage (3 kg/head/day in DM) in a ratio of 75:25. From day 75 to 206, steers were fed the same concentrate with roughage of Napier grass silage (2 kg/head/day in DM) and rice straw (0.9 kg/head/day in DM) in a ratio of concentrate to roughage of 76:24. Cattle were fed twice daily for up to 206 days. The steers were ready for slaughter at an average age of 33 months. Before slaughter, all animals were housed in an enclosure with access to water for 12 h. The animals were weighed, stunned, bled, skinned, eviscerated and washed. Carcasses were split lengthwise and stored at 1 °C for 14 days. Samples of the longissimus thoracis (LT) were taken 14 days post-mortem from the left side of each carcass. All visible fat was removed from the samples, which were then divided into two 3-cm-thick subsamples and two 1.5-cm-thick subsamples. The first 3-cm-thick subsample was used for colour measurement on post-mortem day 14 and then weighed before being vacuum packed and stored at −20 °C to further measure thawing loss, cooking loss, shear force and texture profile. The second 3-cm-thick subsample was vacuum packed, stored at 1 °C for up to 7 days, then unwrapped, meat colour measured 21 days post-mortem, weighed, vacuum packed again, and stored at −20 °C to measure thawing loss, cooking loss, shear force, and texture profile 21 days post-mortem. The first 1.5-cm-thick subsample was vacuum packed and stored at −80 °C to further analyse ribonucleotide content and fatty acid composition on post-mortem day 14. The second 1.5-cm-thick subsample was vacuum packed, stored at 1 °C for up to 7 days, and then stored at −80 °C to further analyse ribonucleotide content on post-mortem day 21.

### 2.3. Meat Characteristics

#### 2.3.1. Colour Measurement

The CIE L*, a*, and b* values of three measurement positions of the cut surface of 3-cm-thick LT steak were measured 14 and 21 days post-mortem after 30 min of blooming at 25 ± 2 °C using a handheld colorimeter with illuminant D65 and 8-mm aperture (MiniScan^®^EZ 45/0 LAV, Hunter Associates Laboratory Inc., Reston, VA, USA).

#### 2.3.2. Thawing Loss, Cooking Loss and Shear Force Analysis

Two 3-cm-thick subsamples, aged 14 and 21 days, were stored at −20 °C for about 2 weeks, thawed at 4 °C for 24 h and then weighed. Weight changes after thawing were expressed as a percentage of thawing loss. Thawed muscle samples were placed in a high-density polyethylene bag before sealing and then placed in a water bath set at 80 °C and boiled for approximately 30 min until they reached an internal temperature of 70 °C. The internal temperature was measured using a thermometer (TM-19475D, Lutron Electronics, Taipei City, Taiwan). The cooked samples were allowed to cool under running tap water for 30 min before being weighed. After cooking, the percentage weight loss was calculated. The cooked samples were sliced parallel to the fibre orientation to obtain eight slices (width × length × height, 1.25 × 1.25 × 3 cm rectangle) before measuring shear force using a texture analyser (Model EZ-SX, Shimadzu, Kyoto, Japan) equipped with a 50 kg load cell and a crosshead speed of 50 mm/min. The shear force value of each sample was averaged from 8 slices.

#### 2.3.3. Texture Profile Analysis

Texture profile analysis (TPA) samples were analysed in the same manner as the shear force samples. After cooling, the cooked surface was removed by cutting with a knife to avoid a hard, dry surface, and each sample was cut into 15-mm cubes. The fibre axis of each cube was perpendicular to the direction of the probe. A texture analyser was used to determine the TPA (model EZ-SX, Shimadzu, Kyoto, Japan). Using a 36-mm-diameter cylindrical probe, each sample was placed between special stainless-steel plates and compressed perpendicular to the muscle fibre orientation in two consecutive cycles of 50% compression (based on sample width, 127 mm/min crosshead speed) with a 1 s pause between cycles. The probe moved downward at a constant speed of 127 mm/min. The force–time data of each test were collected, and the mean values for the TPA parameters of each sample—hardness, springiness, gumminess, chewiness and cohesiveness—were calculated from at least four tests.

### 2.4. Ribonucleotide Analysis

Frozen 1.5-cm-thick subsamples from 14 and 21 days post-mortem were pulverized through cryogenic grinding using a micro-Waring Blender (50–250 mL). One gram of the pulverized muscle sample was homogenized in 6 mL of cold 0.6 M perchloric acid at 23,000 g for 10 s (T25 Ultra-Turrax^®^, Ika, Staufen, Germany) according to [15]. After cooling for 15 min, the homogenate was neutralized with 5.4 mL of 0.8 M KOH and 0.25 mL of KH_2_PO_4_ buffer. The pH of the combined sample was raised to 7 with 0.8 M KOH and the volume was increased to 15 mL with HPLC water. An amount of 1 mL of the supernatant was aspirated into a small tube and frozen at −80 °C after centrifugation at 10,000× *g* for 10 min at 4 °C (Scanspeed 1580R, Labogene, Lillerod, Denmark). The supernatants were analysed by HPLC (Chromaster, Hitachi, Tokyo, Japan) with a UV detector for IMP, inosine, hypoxanthine and GMP after the frozen sample was thawed and centrifuged at 10,000× *g* for 5 min at 4 °C (Scanspeed 1580R, Labogene, Denmark). The stationary phase was a TSK Gel Amide −80 column (Tosoh, Tokyo, Japan) and the elution phase was a buffer of acetonitrile: KH_2_PO_4_, 70:30. External standards were used to calculate ribonucleotide content from a standard curve (57510 inosine-5-monophosphate disodium salt hydrate, 14,125 inosine, H9377 hypoxanthine, and G8377 guanosine-5-monophosphate disodium salt hydrate, Sigma-Aldrich, St. Louis, MO, USA).

### 2.5. Fatty Acid Analysis

The lipid extraction with chloroform was performed as described by [23]. Lipid was extracted from pulverized muscle samples at 14 days post-mortem with chloroform. Methyl Nona decanoate (SFA-013N, Accu Standard, New Haven, CT, USA) was added as an internal standard during the extraction procedure. A fused silica capillary column (100 m × 0.25 mm × 0.2 μm film thickness, model SPTM-2560, Supelco, Bellfonte, PA, USA) was used to evaluate the fatty acid methyl esters (FAM) by gas chromatography (model 7890B, Agilent, Santa Clara, CA, USA). The conditions for gas chromatography were as follows: temperature program: starting temperature 60 °C, followed by an increase of 20 °C/min to 170 °C, 5 °C/min to 220 °C, and 2 °C/min to 240 °C; carrier gas, He; split ratio, 10:1. Fatty acid methyl ester peaks were detected by comparing retention times with authentic standards (F.A.M.E. Mix, C4-C24, Supelco) and measured with an internal standard, nonadecanoic acid (C19:0).

### 2.6. Statistical Analysis

The data were considered as a 3 × 2 factorial arrangement in a completely randomised design, with 3 starch sources (CO, CA, and PI) and 2 ageing periods (14 and 21 days post-mortem). The general linear model procedure (SAS Institute Inc., Cary, NC, USA) was used for analysis of variance to analyse meat quality, texture profile, and ribonucleotide content, including the effects of starch source, ageing period, and their interaction. For fatty acid composition, only starch source was defined as a treatment. The PDIFF option was used to separate least square means. P values less than 0.05 were considered statistically significant. Principal component analysis (PCA) was used to evaluate the relationship between ribonucleotide content and fatty acid composition in relation to the different starch sources and ageing period using XLSTAT software (Addinsoft, Long Island City, NY, USA).

## 3. Results and Discussion

### 3.1. Meat Quality and Texture Profile

In this study, there was no interaction between starch sources and ageing time on meat quality traits (*p* > 0.05), as shown in Table 1. Neither concentrate starch sources nor ageing time had any effect on meat colour (*p* > 0.05) as there were no significant differences in L*, a*, and b* when compared among starch sources or ageing time. There was no significant effect of starch sources on thawing loss and cooking loss (*p* > 0.05). However, ageing time had an effect on thawing loss because thawing loss was higher for a longer ageing time of 21 days than for an ageing time of 14 days, 4.06% and 3.08%, respectively. Shear force was not affected by starch sources and ageing time (*p* > 0.05). Texture profile analysis was performed to investigate whether the starch sources of the concentrate or the ageing time affected the meat texture of LT muscle of dairy steers. No significant interactive effect of starch sources and ageing time on meat texture was found (*p* > 0.05). Neither starch sources nor ageing time had any effect on the meat texture profile (*p* > 0.05). The average hardness of LT muscle in this study was 44.05 N/cm^2^, springiness 0.99 cm, gumminess 24.87 N/cm^2^, chewiness 24.46 N/cm and cohesiveness 0.57.

The fact that no significant effect of starch sources on meat colour, thawing loss, cooking loss, and shear force was observed in the current study could be due to the similar pH of muscle from steers fed different starch sources as mentioned in the study by [7], which used the same sample sources as the current study. According to Hughes et al. 2019 [24], pH was found to be negatively correlated with water holding capacity and lightness. According to Moller et al. (2010) [25], muscle pH was negatively correlated with shear force, implying that shear force tends to decrease as pH increases. Another possible explanation for the non-significant shear force in beef from steers fed different starch sources could be the similar intramuscular fat content, as indicated by the similar amount of total fatty acids in this study. There was a positive correlation between intramuscular fat and tenderness of meat [26].

Proteolysis of muscle fibres can be impaired by ageing, leading to degradation of cytoskeletal proteins and then impairing the ability of muscle fibres to bind water [27]. As muscle structures loosen due to the degradation of myofibrillar and cytoskeletal proteins, the ability to bind water decreases, resulting in a gradual release and drainage of intracellular fluid with prolonged ageing [27]. According to Ledward et al. 1992 [28], the colour of muscle tissue depends on the reflectivity and oxygenation of myoglobin. With ageing, the ability of muscle fibres to retain water decreases, which may improve reflectivity properties and thus increase brightness. Prolonged ageing may also improve the redness of steaks when exposed to oxygen. Ageing may affect mitochondrial function, resulting in decreased competition for oxygen between mitochondria and myoglobin, allowing oxygen to reach tissues [29]. In this study, no effect of ageing on meat colour was observed. This could be due to the fact that the effects of ageing usually occur in the early phase of ageing and myoglobin may not be able to bind oxygen after the early phase. As Colle et al., 2015 [30] found, L* increased post-mortem with longer ageing from 2 to 14 days, but there was no difference in L* during ageing from 14 to 63 days. In the current study, cooking loss was not affected by duration of ageing, but thawing loss was, because a longer ageing of 21 days resulted in higher thawing loss than ageing of 14 days. The higher thawing loss at the longer ageing duration in the present study might be related to the lower ability of the degraded muscle proteins to bind water [27].

Shear force is an objective measurement of tenderness, measured physically by the force required to cut muscle fibres. Shear force has been shown to be inversely related to tenderness. A previous study has shown that tenderness of meat improves when shear force decreases with increasing ageing time [30]. Proteolytic degradation of certain myofibrillar proteins by calpain proteases is responsible for the improved tenderness during ageing [21,31,32]. The presence of the 30 kDa polypeptide and the degradation of troponin-T indicate not only post-mortem proteolysis but also post-mortem decay of the muscle Z-disc [22,31,32]. Duration of ageing had no significant effect on shear force in the present study, which may be due to the fact that little or no post-mortem proteolysis occurred after 14 days of ageing. This is in agreement with the findings of [30] who found that post-mortem ageing of Longissimus lumborum steaks from 2 to 14 days improved tenderness, but no further improvement occurred after 14 days. Ageing also did not affect meat texture in this study as there were no significant differences in hardness, springiness, gumminess, chewiness and cohesiveness of 14-day and 21-day aged beef. Palka, 2003 [33] found that the hardness and chewiness of raw meat was two times lower compared between 5 and 12 days of ageing, but no significant difference was found in cooked meat.

### 3.2. Ribonucleotides

The interaction between starch sources and ageing time on ribonucleotide content was not significant in the present study (*p* > 0.05), as shown in Table 2. There was no significant effect of starch sources on the content of hypoxanthine, inosine and GMP, but there was a significant effect of starch sources on IMP content (*p* < 0.001) as meat from cattle fed with PI had a higher content of IMP than those fed with CO and CA, 107.21, 71.82 and 55.42 mg/100g, respectively. Ageing time affected the content of hypoxanthine, IMP and GMP (*p* < 0.05) but not the content of inosine (*p* > 0.05). At ageing of 21 days, higher hypoxanthine content than ageing for 14 days was found, 34.80 and 27.13 mg/100 g, respectively. At 14 days of ageing, the content of IMP and GMP was higher than at 21 days ageing, 107.44 and 48.86 mg/100 g for IMP and 3.47 and 2.33 mg/100 g for GMP, respectively.

Muscle is known to be turned to meat as food during post-mortem aging. Post-mortem aging improves the flavour and texture of meat. The increase in free amino acids and peptides caused by endogenous proteolytic enzymes in meat during post-mortem aging is associated with improved meat flavour [34]. It is believed that the increase in free amino acids helps to enhance brothy flavour, especially the umami taste, while the increase in peptides is responsible for the mildness of the meat [34]. During post-mortem meat aging, nucleotide triphosphates such as adenosine triphosphate (ATP) and guanosine triphosphate (GTP) are degraded, resulting in umami taste-related compounds such as IMP and guanosine monophosphate (GMP) [19,20]. Adenosine triphosphate is degraded to adenosine diphosphate (ADP) and adenosine monophosphate (AMP), which are subsequently degraded to IMP. Inosine and hypoxanthine are produced once IMP is degraded [19,20]. Inosine is a tasteless substance, but hypoxanthine has a bitter taste [20,35]. Regarding the aging effect, IMP and GMP decreased with increasing hypoxanthine during post-mortem ageing in this study, which is consistent with previous studies [19]. These authors also found a significant decrease in inosine levels during ageing. This differs slightly from this study, which found lower levels of inosine in beef aged 21 days than in beef aged 14 days, with no statistical difference. The lower levels of IMP and GMP and the higher levels of hypoxanthine during ageing in the current study suggest that prolonged ageing has negative effects on beef flavour. However, there are many other factors that affect meat flavour, such as fatty acid composition. Melton et al. (1982) [36] reported that fatty acids such as myristoleic acid, palmitoleic acid, stearic acid, oleic acid, linoleic acid and alpha-linolenic acid can cause good meat flavour.

Propionate production in the rumen and the uptake of glucose from the rumen bypass concentrate in the small intestine are both major sources of glucose in concentrates, while roughage is an important source of acetate produced by the fermentation process in the rumen [5]. Theurer, 1986 [37] reported that dietary ingested starch can reach the small intestine between 4% and 60% in cattle depending on the grain source and processing. Optimizing starch fermentation in the rumen to produce propionate while increasing starch digestion in the small intestine bypassed by the rumen helps improve glucose supply. A very interesting finding of this study is the significantly higher content of the umami substance IMP in LT muscle of dairy steers fed pineapple stem starch in concentrate compared to ground corn and ground cassava as starch sources. This result suggests that cattle fed pineapple stem starch may be more palatable than the other two starch sources. The higher content of IMP might be related to the higher content of its precursor, glucose, in PI fed steers. Khongpradit et al., 2020 [15] found a significantly higher content of Ruminococcus bromii C1 and total short chain fatty acids (SCFA) in the rumen of cattle fed PI than CO and CA, suggesting that PI is more fermented in the rumen, which could be related to the smaller particle size of starch, lower neutral detergent fibre (NDF) and lower crude protein and lipid content associated with starch, making this starch source more degradable and fermentable than others. However, they found that the percentage of propionic acid in the rumen fermentation profile, the main source of glucose production, did not differ between starch sources. Therefore, the possible explanation for the higher IMP content in PI could be related to the higher glucose supply through small intestinal digestion. In a previous study, significantly higher amylose content was found in pineapple stem starch than corn and cassava [14]. Goats fed high amylose corn in the total mixed ration had more starch that was not degradable in the rumen and therefore more starch entered the small intestine for digestion, resulting in higher blood glucose levels [38]. Further studies are needed on the effects of the different starch sources in this study that could affect blood glucose levels.

### 3.3. Fatty Acid Composition

The fatty acid composition of the different starch sources in the concentrate is shown in Table 3. There was no effect of starch source on fatty acid composition except that cattle fed CO had the highest oleic acid but the lowest erucic acid (*p* < 0.05), in contrast to cattle fed PI, which had the lowest oleic acid but the highest erucic acid. The content of oleic acid was highest in the meat of cattle fed CO and lowest in cattle fed PI, while cattle fed CA were not significantly different from the others, 43.70, 42.36 and 40.65% of total fatty acids, respectively. The content of erucic acid was higher in PI than the others, 0.45, 0.47 and 0.74% of total fatty acids for cattle fed CO, CA and PI, respectively. In addition, cattle fed PI tended to have more palmitic acid than the others (P = 0.092), 27.8, 27.90 and 29.59% of total fatty acids for meat from cattle fed CO, CA and PI, respectively. The most abundant fatty acid in the meat of this study was monounsaturated oleic acid, followed by saturated palmitic acid and stearic acid, which ranged from 40.64 to 43.70, 27.81 to 29.59, and 10.68 to 11.39% of total fatty acids, respectively. Starch sources had no effect on MUFA, PUFA (polyunsaturated fatty acid) and SFA (saturated fatty acid) (*p* > 0.05). However, meat from cattle fed PI tended to have lower levels of desirable fatty acids (DFA), which include MUFA, PUFA and stearic acid, than others.

Feeding cereal grains is one way to improve net energy supply because energy-rich grains can be used to form volatile fatty acids and glucose in the rumen and small intestine. When IMF preferentially uses glucose as a substrate for fatty acid synthesis while subcutaneous fat prefers acetate, the deposition of IMF is greater in diets with higher concentrate content as explained by [39]. According to Park et al. (2018) [5], the uptake of excess net energy is a key component of intramuscular fat (IMF) deposition. Konpradit et al. (2020) [15] reported the higher degradability of PI starch in the rumen resulting in higher weight gain, average daily gain and feed conversion ratio in PI-fed steers compared to CO- and CA-fed steers. However, the different sources of starch in the concentrate ration did not affect the intramuscular fat content, as indicated by the similar amount of total fatty acids in the present study. This is in agreement with Kongpradit et al., 2021 (inpress) [7] who reported non-significant intramuscular fat content in the meat of cattle fed different starch sources of CO, CA and PI. Regarding the fatty acid composition in the intramuscular fat of cattle, the concentration of MUFA in adipose tissue is catalysed by the enzyme Δ9-desaturase (stearoyl-CoA desaturase) [40]. Palmitic acid and stearic acid are the preferred substrates that are converted to palmitoleic acid and oleic acid, respectively [41]. The higher concentration of palmitic acid and the lower concentration of oleic acid in the intramuscular fat of meat from steers fed PI in the present study might be due to the lower activity of Δ9-desaturase. Therefore, further studies to determine Δ9-desaturase activity in the meat of cattle fed PI are needed to understand how PI affects lipogenesis. The other possible explanation for the higher oleic acid in the meat of cattle fed CO, and the lower content in the meat of cattle fed PI could be due to the energy balance between the different starch sources of the concentrate, which is why a higher proportion of rice bran was used in the CA and PI concentrate diets than in the CO concentrate diet, as detailed in Supplementary Table S1 [15]. Since corn contains a higher percentage of linoleic acid than rice bran (56.5% and 34.8%, respectively), as mentioned by [35], this fatty acid would be converted to oleic acid by biohydrogenation in the rumen and then accumulate in the muscles of cattle [36].

Grain-fed beef is one of the most plentiful sources of MUFAs in the form of oleic acid and may be a significant source of MUFAs in the human diet (18:1, n-9) [37]. The importance of MUFAs in cardiovascular health has been extensively established. Higher oleic acid content in beef is beneficial as it can increase blood HDL cholesterol levels and reduce cardiovascular disease risk factors [12]. In the present study, it was found that the significantly lower oleic acid content, a trend toward lower DFA content and a trend toward higher palmitic acid content in the meat of cattle fed PI could be a risk factor for human health. However, previous studies suggest that the oleic acid content of grain-fed diets is higher than that of grass-fed beef [13,37], including the concentrate base in this study. The oleic acid content in the meat of cattle fed PI is still higher compared to natural grass-fed beef and is therefore healthier.

### 3.4. Ribonucleotide Content and Fatty Acid Composition in Relation to Different Starch Sources in Concentrate and Ageing Period

**Figure 1:**
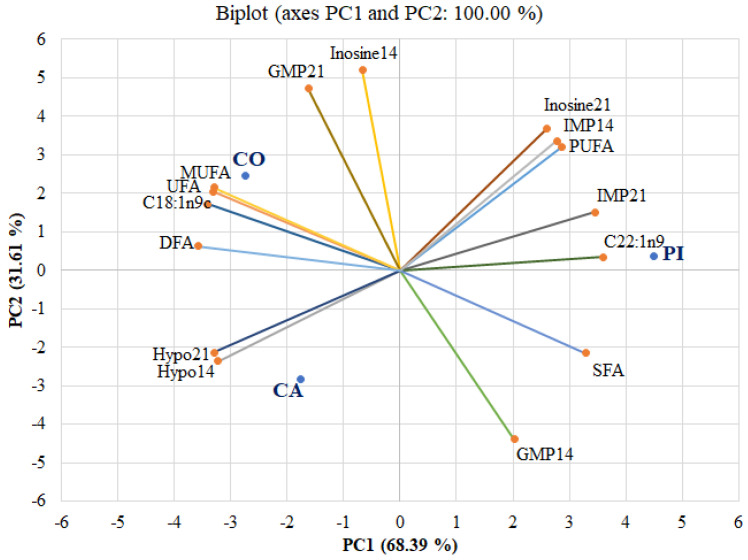
The results of principal component analysis (PCA) of ribonucleotide content and fatty acid composition at different ageing times varied among the different starch sources: CA = ground cassava, CO = ground corn, PI = pineapple stem starch, SFA (saturated fatty acids), MUFA (monounsaturated fatty acids), PUFA (polyunsaturated fatty acids), UFA (unsaturated fatty acids), DFA (desirable fatty acids), Hypo14 or Hypo21 (hypoxanthine content measured at day 14 or 21 post-mortem), inosine14 or inosine21 (inosine content measured on day 14 or 21 post-mortem), IMP14 or IMP21 (inosine monophosphate content measured on day 14 or 21 post-mortem), GMP14 or GMP21 (guanosine monophosphate measured on day 14 or 21 post-mortem).

PCA was performed to evaluate the relationships between ribonucleotide content and fatty acid composition of LT muscle from dairy steers fed different starch sources and aged for different periods of time, 14 days or 21 days (Figure 1). There were two principal components (PC1 and PC2) that explained 100% of the total variance (68.39% and 31.61%, respectively). The first component, PC1, was strongly positively loaded by inosine at 21 days of ageing, IMP at 14 days and 21 days of ageing, PUFA, SFA and C22:1n9, but strongly negatively loaded by hypoxanthine content at 14 days and 21 days of ageing and MUFA, UFA, DFA and C18:1n9c content. The second component, PC2, was strongly positively loaded by GMP content at 21 days ageing and inosine content at 14 days ageing but was strongly negatively loaded by GMP content at 14 days ageing.

The PCA bi-plot showing ribonucleotide content and fatty acid composition at different ageing times varied among the different starch sources. The different starch sources exhibited different characteristics. It was found that CO was strongly associated with MUFA, UFA, DFA and C18:1n9c content. Conversely, PI was strongly associated with fatty acid composition, PUFA, SFA, C22:1n9 and ribonucleotide content; inosine at 21 days of ageing and IMP at 14 and 21 days of ageing. CA showed significant differences in fatty acid composition and ribonucleotide content from others, especially hypoxanthine at 14 and 21 days of ageing. From the PCA bi-plot, the use of CO as a starch source in concentrate may provide healthier beef as it is positively strongly associated with the content of MUFA, UFA and DFA, while the use of PI as a starch source may provide tastier beef, as it is positively strongly associated with the content of IMP.

## 4. Conclusions

Pineapple stem starch can be used as an alternative starch source in cattle concentrates without affecting meat quality, and it can also reduce feed costs. PI may also improve meat flavour, as meat from cattle fed PI may have a higher content of IMP than meat from cattle fed CO and CA. However, the meat from cattle fed CO appears to be healthier as it contains more oleic acid than others. The optimum ageing time to improve meat quality in dairy steers could not be more than 14 days because the improvement in meat quality did not occur after 14 days of ageing, and a longer ageing time could have a negative effect on meat flavour because the IMP and GMP content is lower after 14 days of ageing with higher hypoxanthine content.

## Figures and Tables

**Table 1:** Effect of starch source of concentrate and ageing period on meat quality and texture profile of fattening dairy steers.

Trait	Treatment (T) ^1^			Ageing (A)		RMSE ^2^	*p*-Value		
	CO	CA	PI	14 Days	21 Days		T	A	T × A
Meat Quality									
Colour									
L*	35.32	36.51	36.41	35.67	36.49	2.32	0.347	0.268	0.933
a*	14.46	15.29	14.78	14.96	14.72	1.79	0.509	0.676	0.339
b*	41.36	42.13	42.24	41.44	42.36	3.21	0.739	0.358	0.641
Thawing loss (%)	3.31	3.48	3.93	3.08 ^b^	4.06 ^a^	1.18	0.430	0.019	0.777
Cooking loss (%)	19.23	21.01	21.73	19.60	21.72	4.22	0.287	0.121	0.712
Shear force (kg)	5.72	4.97	5.03	5.41	5.07	1.08	0.151	0.328	0.949
Texture Profile									
Hardness (N/cm^2^)	40.57	42.74	48.85	40.92	47.19	22.80	0.615	0.392	0.890
Springiness (cm)	0.99	0.99	0.99	0.99	0.99	0.00	0.473	0.694	0.636
Gumminess (N/cm^2^)	24.29	23.95	26.38	22.98	26.76	12.60	0.863	0.351	0.931
Chewiness (N/cm)	23.48	23.94	25.95	22.27	26.64	12.46	0.858	0.275	0.945
Cohesiveness(ratio)	0.57	0.59	0.55	0.57	0.56	0.06	0.206	0.880	0.980

^1^ CO, ground corn; CA, ground cassava; PI, pineapple stem starch. ^2^ root mean square error; ^a,b^ Lsmeans having different superscripts within the same main effect are significantly different (*p* < 0.05).

**Table 2:** Effect of starch source of concentrate and ageing period on ribonucleotide of fattening dairy steers.

Trait ^1^	Treatment (T) ^2^			Ageing (A)		RMSE ^3^	*p*-Value		
	CO	CA	PI	14 Days	21 Days		T	A	T x A
Hypo ^4^	31.76	33.52	27.61	27.13 ^d^	34.80 ^c^	9.80	0.294	0.019	0.507
Inosine	39.18	36.43	38.06	40.31	35.47	12.12	0.848	0.216	0.900
IMP ^5^	71.82 ^b^	55.42 ^b^	107.2 ^a^	107.44 ^a^	48.86 ^b^	35.20	0.002	<0.0001	0.629
GMP ^6^	2.49	3.09	3.10	3.47 ^c^	2.33 ^d^	1.67	0.552	0.039	0.079

^a,b^ Lsmeans having different superscripts within the same main effect are significantly different (*p* < 0.01).; ^c,d^ Lsmeans having different superscripts within the same main effect are significantly different (*p* < 0.05).; ^1^ mg/100 g.; ^2^ CA = ground cassava; CO = ground corn; PI = pineapple stem starch.; ^3^ root mean square error.; ^4^ hypoxanthine.; ^5^ inosine monophosphate.; ^6^ guanosine monophosphate.

**Table 3:** Effect of starch source of concentrate on fatty acid composition of fattening dairy steers.

Trait		Starch Source ^1^			RMSE	*p*-Value
		CO	CA	PI		
Fatty Acid Composition (% of Total Fatty Acids)						
Lauric acid	C12:0	0.18	0.19	0.17	0.05	0.776
Myristic acid	C14:0	4.37	4.84	4.83	0.46	0.133
Myristoleic acid	C14:1	1.55	1.81	1.54	0.45	0.501
Pentadecylic acid	C15:0	0.25	0.25	0.24	0.08	0.961
Palmitic acid	C16:0	27.81	27.90	29.59	1.57	0.092
Palmitoleic acid	C16:1	6.28	6.23	6.10	0.81	0.917
Margaric acid	C17:0	0.57	0.55	0.60	0.08	0.477
Heptadecenoic acid	C17:1	0.55	0.51	0.56	0.11	0.657
Stearic acid	C18:0	10.93	11.39	10.68	1.38	0.650
Oleic acid	C18:1n9c	43.70 ^a^	42.36 ^ab^	40.65 ^b^	1.91	0.027
Linoleic acid	C18:2n6c	1.15	1.10	1.18	0.24	0.821
α-Linolenic acid	C18:3n3	0.23	0.23	0.18	0.07	0.383
Heneicosylic acid	C21:0	0.24	0.23	0.19	0.10	0.580
Erucic acid	C22:1n9	0.45 ^b^	0.47 ^b^	0.74 ^a^	0.10	0.021
Arachidonic acid	C20:4n6	0.15	0.18	0.19	0.07	0.659
Lignoceric acid	C24:0	0.11	0.13	0.17	0.08	0.413
Nervonic acid	C24:1	1.49	1.64	2.40	0.82	0.116
Total fatty acid ^2^		8.60	9.37	9.07	1.82	0.748
SFA		44.45	45.49	46.45	1.99	0.200
MUFA		54.02	53.01	51.99	2.02	0.202
PUFA		1.53	1.51	1.55	0.27	0.956
UFA		55.55	54.51	53.55	1.99	0.200
DFA		66.48	65.91	64.22	1.76	0.072

^a,b^ Lsmeans having different superscripts within the same main effect are significantly different (*p* < 0.05).; ^1^ CA = ground cassava; CO = ground corn; PI = pineapple stem starch.; ^2^ g/100g muscle.; SFA (saturated fatty acids): C12:0+C14:0+C15:0+C16:0+C17:0+ C18:0+C21:0+C24:0; MUFA (monounsaturated fatty acids): C14:1+C16:1 + C17:1 + C18:1n9c+C22:1+C24:1; PUFA (polyunsaturated fatty acids): C18:2n6c + C18:3n3 + C20:4n6; UFA (unsaturated fatty acids): MUFA + PUFA; DFA (desirable fatty acids): MUFA + PUFA + C18:0.

## Data Availability

The data presented in this study are available on request from the corresponding author.

## Supplementary Materials

The following are available online at www.mdpi.com/article/10.3390/foods10102319/s1, Table S1: Ingredients and nutrient composition of concentrates and roughage (% DM).
