# Supplementary material for: The Effects of Using Pineapple Stem Starch as an Alternative Starch Source and Ageing Period on Meat Quality, Texture Profile, Ribonucleotide Content, and Fatty Acid Composition of Longissimus Thoracis of Fattening Dairy Steers
Source: Foods. 2021 Sep 29;10(10):2319. doi: 10.3390/foods10102319 (PMC8535452; doi:10.3390/foods10102319)
Supplement: Supplementary file 1 [file foods-10-02319-s001.zip › foods-1391671-supplementary.pdf]

foods MDPI

Table S1. Ingredients and nutrient composition of concentrates and roughage (% DM) [15].

**Item** (% **DM**)	**Concentrate** [1]			**Roughage**	
	**CO**	**CA**	**PI**	**Napier Grass Silage**	**Rice Straw**
Ingredient					
Ground corn	40.24	-	-		
Ground cassava	-	40.28	-		
Pineapple stem starch	-	-	40.23		
Soybean meal	6.75	6.62	6.99		
Rice bran	-	13.38	13.35		
Defatted rice bran	16.25	2.81	2.81		
Defatted palm kernel meal	26.06	25.55	25.50		
Molasses	7.83	7.68	7.66		
Urea	0.16	1.04	1.20		
Vitamin and mineral mixture [2]	0.52	0.52	0.52		
Sulphur	0.11	0.10	0.10		
Salt	0.32	0.31	0.31		
Dicalcium phosphate	1.24	1.21	1.21		
Sodium bicarbonate	0.52	0.51	0.50		
Nutrient composition					
Dry matter (%)	87.06	87.46	87.74	20.42	91.30
Crude protein	16.79	16.34	16.28	6.32	5.90
Ether extract	3.13	3.25	3.17	1.71	-
Ash	6.91	8.68	6.60	11.37	-
NDF	40.81	38.45	37.74	73.20	74.00
ADF	31.07	27.99	27.54	55.03	44.50
Calcium	0.84	0.90	0.63	0.27	-
Phosphorus	0.80	0.63	0.63	0.24	-
Total carbohydrates	73.17	71.73	73.95	-	-
Starch	37.54	38.63	38.70		

Total carbohydrates = 100—(% crude protein + % ether extract + % crude ash).; [1]CO, ground corn; CA, ground cassava; PI, pineapple stem.; [2]Agromix beef No. 46: vitamin A = 2,160,000 IU, vitamin B3 = 100,000 IU, vitamin E = 5,000 IU, Mn = 8.5 g, Zn = 6.4 g, Cu = 1.6 g, Mg = 16 g, Co = 320 mg, I = 800 mg, Se = 32 mg (Agromix beef No.46: A.I.O. Co. LTD, Nakhon Pathom, Thailand).
